# Supplementary material for: Integrating biomedical research and electronic health records to create knowledge-based biologically meaningful machine-readable embeddings
Source: Nat Commun. 2019 Jul 10;10:3045. doi: 10.1038/s41467-019-11069-0 (PMC6620318; doi:10.1038/s41467-019-11069-0)
Supplement: Supplementary file 1 — Supplementary Information New [file 41467_2019_11069_MOESM1_ESM.pdf]

# SUPPLEMENTARY INFORMATION

# Integrating biomedical research and electronic health records to create knowledge-based biologically meaningful machine-readable embeddings

**Charlotte A. Nelson, Atul J. Butte, Sergio E. Baranzini**

# Table of Contents

***SUPPLEMENTARY INFORMATION*** ........................................ ***1***

**Supplementary Note1** ........................................ **3**

Inferring *Disease-Disease* Relationships from PSEVs ........................................ 3

Recovering Deleted *Disease* Resembles *Disease* Relationships ........................................ 3

*Compound* Benchmark ........................................ 4

SideEffect to Anatomy Benchmark ........................................ 7

**SUPPLEMENTARY FIGURES** ........................................ **11**

Supplementary Figure 1. Calculating non-uniform probability of random jump. ........................................ 12

Supplementary Figure 2. BMI PSEVs created using continuous BMI contain phenotypic and genotypic information. ........................................ 14

Supplementary Figure 3. PSEVs embed first neighbors in SPOKE and learn new relationships ........................................ 16

Supplementary Figure 4. Recovering deleted *Disease-Disease* edges ........................................ 18

Supplementary Figure 5. Recovering deleted *Compound-Gene* edges ........................................ 20

Supplementary Figure 6. Recovering deleted *Compound-Compound* edges. ........................................ 22

Supplementary Figure 7 MEDLINE Anatomy-SideEffect Relationships are Top Ranked Nodes in PSEV. ........................................ 24

**Supplementary Tables** ........................................ **26**

Supplementary Table 1. **SPOKE nodes**. Source(s) and counts of each node type in SPOKE. ........................................ 27

Supplementary Table 2. **SPOKE edges.** Source(s) and counts of each edge label in SPOKE. ........................................ 28

**Supplementary References** ........................................ **29**

## Supplementary Note 1

### Inferring *Disease-Disease* Relationships from PSEVs

Utilizing the normalized original matrix (PSEV), benchmark matrix ($PSEV^{\Delta DD, \Delta DG}$) and the three random PSEV matrices, we checked to see if the deleted SPOKE *Disease*-RESEMBLES_DrD-*Disease* edges could be inferred directly from the PSEV matrices. The *Disease*-RESEMBLES_DrD-*Disease* edges in SPOKE were derived using MEDLINE co-occurrences (n=1,086). This evaluation mirrors that used to test the recovered *Disease-Gene* relationships. However, in this case the *Disease*s elements (n=129 using Diseases that resemble at least one other Disease or n=137 for entire set of Diseases in SPOKE) in each *Disease* PSEV were ranked such that the one ranked 1 would denote the most similar to a given *Disease*. All PSEV matrices were evaluated using this method (Supplementary Figure 4).

### Recovering Deleted *Disease* Resembles *Disease* Relationships

We next used PSEV to create a *Disease-Disease* network ($DD^{PSEV}$) as we did the *Disease-Gene* networks and used a similar strategy to build background networks as comparators ($DD^{PSEV\Delta DD, \Delta DG}$, $DD^{RANDOM}$, $DD^{SPOKE\ SHUFFLE}$, and $DD^{SEP\ SHUFFLE}$) using the original, benchmark and three random PSEV matrices. These *Disease-Disease* networks were then evaluated by the number of edges they shared with the *Disease*-RESEMBLES_ *Disease*_(DrD)-network from SPOKE ($DD^{SPOKE}$). The RESEMBLES_DrD edges in SPOKE were created using the most statistically significant MEDLINE term co-occurrences[1] (n=1,086, p<0.005, $\chi^2$). Again, we found that $DD^{PSEV}$ (and even $DD^{PSEV\Delta DD, \Delta DG}$) was able to recover more of the deleted edges (on average 4.7x and 3.7x accordingly) than any of the three random networks (Supplementary Figure 4B).

Interestingly, one of the three random networks ($DD^{SPOKE\ SHUFFLE}$) performed significantly better than the other two. We hypothesize this is due to the fact that some *Disease-Disease* relationships are observable in the EHRs as co-morbidities and misdiagnoses. This information is then feed directly into the *Disease* SEPs, making *Disease*s that resemble other *Disease*s significant in the PSEVs. Since this relationship does not always need to traverse paths in SPOKE, it is observable in the $DD^{SPOKE\ SHUFFLE}$. In contrast, in $DD^{SEP\ SHUFFLE}$ the altered mappings between the SEPs and SPOKE disrupt observable relationships in the EHRs, which in turn inhibits the prioritization of *Disease*

nodes. These results highlight the accuracy of the mappings between EHR concepts to nodes in SPOKE.

Additionally, in order to learn how we are able to correctly identify related *Diseases* even after deleting *Disease-Gene* and *Disease-Disease* edges from SPOKE, we retraced the shortest paths between significant SEPs of a given *Disease* to its target related *Disease(s)*. Figure 2A shows how Hypertension was ranked as a top *Disease* in the Type 2 Diabetes $PSEV^{\Delta DD, \Delta DG}$. The pressure from the EHRs of Type 2 Diabetes patients pushes the flow of information to the *Anatomy* in which Hypertension is localized, *Symptoms* presented by Hypertension, and *Compounds* that treat or palliate Hypertension. This flow of information makes Hypertension a top ranked *Disease* for Type 2 Diabetes. Further, this pattern of information flow, particularly through *Anatomy* and *Symptom* nodes, is very conserved in the shortest paths between *Disease* pairs.

## *Compound* Benchmark

### *Compound-Compound PSEV Based Network*

We created *Compound* benchmark PSEVs ($PSEV^{\Delta CC, \Delta CG}$) by removing the *Compound-Compound* and *Compound-Gene* relationships in SPOKE prior to PSEV creation. We then used z-scores to normalize the $PSEV^{\Delta CC, \Delta CG}$.

### *Random Compound PSEVs*

The three random *Compound* PSEV matrices were derived in the same way as the random *Disease* PSEV matrices. First, $PSEV^{RANDOM}$ was created by permuting the nodes in the *Compound* PSEVs using the Fisher–Yates method. Second, $PSEV^{SPOKE\ Shuffle}$ was created by shuffling the edges within SPOKE, by edge type. Third, $PSEV^{SEP\ Shuffle}$ was created by shuffling the edges between SEPs and SPOKE, by edge type. Neither *Compound-Compound* or *Compound-Gene* edges were deleted prior to random PSEV calculation. All random PSEV matrices were then z-score normalized.

### *Inferring Compound-Protein binding partners using EHR embeddings.*

Employing the original matrix (PSEV), benchmark matrix ($PSEV^{\Delta CC, \Delta CG}$) and three random matrices ($PSEV^{random}$, $PSEV^{shuffled\_SPOKE}$, and $PSEV^{shuffled\_SEP}$) we tested whether the molecular targets of a given compound were ranked higher in that *Compound*'s PSEV. To test this we used the

*Compound*-BINDS_CbG-*Gene* edges in SPOKE which were derived from a *Compound*'s protein targets from BindingDB [2,3], DrugBank [4,5], and DrugCentral [6] (11,571 edges).

Though this method of evaluation is very similar to the previous methods, it differed in that we selected a fixed number of top K ranked nodes to select from each *Compound* PSEV (K=150). The decision to choose a fixed K was based on the fact that the average number of Gene binding partners per Compound was much smaller than the average number of Genes that associate with Diseases. The value of K was calculated by finding the point at which the patient population no longer contributes positively to the rank of the target *Gene*. The simplest way to calculate patient contribution to the target *Gene* is through proportion of patients on a given *Compound* that have been diagnosed with a *Disease* that is related to the target Gene (Supplementary Fig 3C). This is computed by z-score normalizing the transition probability matrix and summing the values of *Diseases* that are related to the target *Gene* for a given *Compound*. We then plot the aggregated z-scores against rank to find the point in which the aggregated z-scores reaches zero (K=150; Supplementary Fig 3C).

Interestingly, we found that the most significant negative information flow (right end of the plot) was associated with the worst ranked *Genes* and often corresponded to contraindications. For example, Tolmetin, a non-steroidal anti-inflammatory drug, targets *PTGS1* - a gene known to be related to hypertension[7, 8] (Supplementary Fig 3A). However, Tolmetin is contraindicated for hypertension because it increases the risk of adverse cardiovascular events. As a result, within the population of patients that were prescribed Tolmetin, the number of patients that were also diagnosed with hypertension was fewer than expected. This causes negative information flow through *PTGS1* in the Tolmetin PSEV.

Next, selecting the top 150 *Genes* per *Compound PSEV*, we built *Compound-Gene* networks using the original ($CG^{PSEV}$), benchmark ($CG^{PSEV\Delta CC,\ \Delta CG}$), and three random PSEV matrices ($CG^{RANDOM}$, $CG^{SPOKE\ SHUFFLE}$, and $CG^{SEP\ SHUFFLE}$) respectively. We then compared the number of overlapping edges between the $CG^{SPOKE}$, a *Compound-Gene* network created with the *Compound*-BINDS_CbG-*Gene* edges in SPOKE, and the other CG networks. When selecting the top K *Genes* using only *Genes* that have at least one BINDS_DbG edge, we found that $CG^{PSEV\Delta CC,\ \Delta CG}$ and $CG^{PSEV}$ shared on average 1.9x and 6.9x more edges than the three random networks (Supplementary Fig. 3B) and when selecting the top K from all Gene nodes in SPOKE, the sharing increased to 4.3x and 51.5x

respectively (Supplementary Fig. 3B insert). These results show that adding patient information from the EHRs enables the discovery of Compound-Gene relationships in SPOKE.

Finally, to unravel how *Compound* binding partners are highly ranked in PSEVs even after *Compound-Gene* and *Compound-Compound* edges are deleted, we again retraced the shortest paths between significant SEPs and the target *Gene*. Ursodeoxycholic acid is a cholesterol-lowering medication that can also be used to dissolve gallstones and treat liver disorders and is known to target the protein ABCB11, a member of the superfamily of ATP-binding cassette (ABC) transporters[9, 10, 11]. Supplementary Figure 5A shows how EHRs from patients prescribed Ursodeoxycholic acid guide the flow of information to ABCB11. The information is driven towards *BiologicalProcess* and *Pathway* nodes that ABCB11 participates in and *Diseases* that are localized in *Anatomies* that ABCB11 is expressed or regulated in. Since *Gene* nodes only represent a small fraction of SEPs, this pattern of flow from SEP to target *Gene* is not very common because it includes a *Gene* node (gamma-glutamyltransferase 1, *GGT*) as one of the SEPs. High levels of GGT are often associated with liver or bile duct diseases, which explains why patients may benefit from this drug, as well as informs the connection to ABCB11. More commonly, the shortest paths will involve information flow through *Disease, Anatomy*, and occasionally *Gene* nodes.

### *Compound fingerprint similarity in EHR embeddings.*

Analogous to generating the *Disease-Disease* networks, we created *Compound-Compound* networks using the top K ranked *Compound* nodes in the original ($CC^{PSEV}$), benchmark ($CC^{PSEV\Delta CC,\Delta CG}$), or random PSEV ($CC^{RANDOM}$, $CC^{SPOKE\ SHUFFLED}$, and $CC^{SEP\ SHUFFLED}$) matrices, where K equals the number of similar Compounds to a selected Compound. Then we created a fingerprint-based *Compound-Compound* network ($CC^{SPOKE}$) using the *Compound*-RESEMBLES_CrC-*Compound* edges (n=7,703) in SPOKE. The *Compound*-RESEMBLES_CrC-*Compound* edges in SPOKE were derived using the similarity between two Compounds extended connectivity fingerprints[12, 13] and filtered based on their Dice coefficient[14, 1]. Next, we computed the number of edges that were shared between $CC^{SPOKE}$ and the other *Compound-Compound* networks. We found that the observed number of shared edges in $CC^{PSEV\Delta CC,\Delta CG}$ and $CC^{PSEV}$ were on average significantly higher than random (4.4x and 15.2x) when selecting from the set of Compounds that resembles at least one other Compound and even higher (4.9x and 17.6x) when selecting from the entire set of nodes in SPOKE (Supplementary Figure 6B). Again the p-values in the figure were calculated using Fisher's

method to combine the p-values for selecting the top K *Compounds* from each *Compound* $PSEV^{\Delta CC, \Delta CG}$.

Just as when we inferred *Disease-Disease* relationships, we noticed that $CC^{SPOKE\ SHUFFLED}$ performed better than the other two random networks. Again, this is likely because we attempted to predict relationships that can sometimes be observed without traversing SPOKE because they are observable in the EHRs. Therefore, shuffling the edges within SPOKE won't greatly impact this prediction. Furthermore, these results also demonstrate that we are correctly mapping medication orders in the EHRs to *Compound* nodes in SPOKE.

To elucidate how the benchmark PSEVs could infer whether two compounds were similar, we again found the shortest paths between the important SEPs and target (*Compound*) node. We found that in order to connect Compounds, the random walker usually followed one of two path patterns. In one pattern, the information from the patient population on a given *Compound* is "pushed" through shared *SideEffects* and *PharmacologicalClasses*. For example, Tioconazole resembles Sertaconazole (similarity=0.80) and in order to connect the two Compounds pressure from patients on Tioconazole must move information flow through the *SideEffects* Pruritus, Erythema, Dry skin, and Application site reaction and the *PharmacologicalClass* Azoles (Supplementary Fig. 4A left). The other shortest path pattern for recovering similar *Compounds* is observed when two *Compounds* treat the same *Disease*. An example of this is seen when connecting Trihexyphenidyl to Procyclidine (similarity=0.98; Supplementary Fig. 4A right) which both are used to treat Parkinson's disease (PD). Here, most of the weight from the EHRs of patients on Trihexyphenidyl is coming from PD and nodes related to PD: Trihexyphenidyl (*Compound* treats PD), Dyskinesias (*Symptom* presented by PD), and Tremor (*Symptom* presented by PD). This results in significant information flow to the Procyclidine node. These results prove the PSEVs ability to identify Compounds with similar structures as well as illustrate what components of the EHRs and relationships of SPOKE are most critical to inform that decision.

## SideEffect to Anatomy Benchmark

### *MEDLINE Co-occurrence Gold Standard*

MEDLINE yearly publishes the co-occurrences of MeSH terms found on Pubmed publications. After converting *Anatomy* and *SideEffect* identifiers to MeSH IDs we created a counts matrix for co-occurring *Anatomy* and *SideEffect* terms. Out of the 699,745 possible pairs,

222,224 had at least one co-occurrence). Then we preformed $\chi^2$ to determine the significance of the *Anatomy-SideEffect* MEDLINE relationships. Since 51% of relationships had a p-value less than or equal to 0.05, we decided to strengthen the filter to the top 5% of p-values (p=7.4E-75, $\chi^2$) leaving 11,112 *Anatomy-SideEffect* pairs.

### *PSEV Benchmark Anatomy-SideEffect Network*

First, we used z-score to normalize the PSEV matrix. Then we transposed the PSEV matrix ($PSEV^T$) to obtain a vector (n=3,233) for every node in SPOKE. This vector describes the importance of a given SPOKE node for each SPOKE Entry Point (SEPs). Next, vectors from $PSEV^T$ were then used to calculate the cosine similarity between *Anatomy* and *SideEffect* nodes. Finally, the similarities were ranked (1 to 699,745), such that a rank of 1 signified the most similar *Anatomy-SideEffect* pair in the matrix.

### *Random Anatomy-SideEffect Networks*

To create a random $PSEV^T$ matrix, the normalized benchmark $PSEV^T$ was shuffled using the Fisher–Yates method to randomly permute the rows of the matrix. The random PSEV matrix was then used to calculate the cosine similarity between the *Anatomy-SideEffect* pairs and ranked from 1 to 699,745 in the same way as the benchmark matrix.

### *Overlapping Anatomy-SideEffect Links*

Benchmark and random *Anatomy-SideEffect* networks were created using the top k (k=1 to 699,745, increasing in intervals of 5%) nodes in PSEV and $PSEV^{RANDOM}$ accordingly. Supplementary Figure 7 shows the overlapping counts and fraction between the RP networks and the 11,112 *Anatomy-SideEffect* pairs from MEDLINE. Inserts in Supplementary Figures 7A-C focus on k<= 11,112, corresponding the number of *Anatomy-SideEffect* pairs from MEDLINE. The highest fold changes 18.1 over random occurred in the top k=1,000 respectively (Supplementary Figure 7C insert).

### *Recovering the major shortest paths between SideEffect and Anatomy nodes*

First, we needed to find the nodes that contributed most weight to the similarity of the S*ideEffect- Anatomy* pair. Since we used cosine similarity, which is equivalent to the dot product of two unit vectors, we simply multiplied the *SideEffect* and *Anatomy* transposed PSEVs and

selected the highest 0.1% of nodes. Those nodes are labeled as top contributors in Supplementary Figures 7D-F. We then found the shortest paths between each top contributor node and the target *SideEffect* and *Anatomy* nodes.

### *SideEffect-Anatomy relationships in embedded EHR concepts match MEDLINE co-occurrences.*

Although it is natural to draw a connection between drug side effects and the anatomies they affect (e.g. a headache must somehow relate to the brain), *SideEffect* and *Anatomy* nodes are not directly connected in SPOKE. In fact, in order to get from a *SideEffect* to an *Anatomy* node one must traverse a minimum of three edges. As a result, correctly inferring the relationships between *Anatomy* and *SideEffect* nodes would show that appropriate weights are assigned to distant nodes in the network. To test this, we created a gold standard *SideEffect-Anatomy* network using only highly significant relationships from MEDLINE co-occurrences ($SeA^{MEDLINE}$) (p=7.4e-75, $\chi^2$; n=11,112; avg 6.4 Anatomy per SideEffect). Next, we computed a *SideEffect-Anatomy* cosine similarity matrix using the transposed PSEV matrix (See methods). We then selected the most similar *SideEffect-Anatomy* pairs to create a PSEV-based *SideEffect-Anatomy* network ($SeA^{PSEV}$). These relationships were also tested against a random network ($SeA^{RANDOM}$) that was generated by permuting each PSEV, as in the $DD^{RANDOM}$ networks (Supplementary Figure 7).

In the first interval (k=1000), we observed 18.1 times more overlapping edges than expected by chance (Supplementary Figure 7C insert; binomial p value = 9.7E-251). By accurately ranking the relationships between *SideEffect* and *Anatomy* nodes, we further demonstrate that PSEVs are a valid strategy to infer missing links in SPOKE. This result is even more consequential given that *SideEffect* and *Anatomy* nodes are far away in SPOKE.

Similar to before when we found the shortest paths between SEPs and the target node to understand how deleted edges where recovered, we wanted to find the paths that enabled us to learn relationships between *SideEffect* and *Anatomy* nodes. To achieve this, we found the nodes in the transposed PSEVs that contributed the most to the *SideEffect* and *Anatomy* similarity. We then looked at the shortest paths between those nodes and the target *SideEffect* and *Anatomy* nodes. Supplementary Figures 5D-F show examples of these paths. The first example shows how Aggression connects to locus coeruleus (LC), a part of the brain that is involved in emotions, arousal, attention, and stress response [15]. The nodes that contribute the most to the similarity are *Compounds* and all have the *SideEffect* Aggression. Additionally, those *Compounds* bind or

regulate *Genes* expressed or regulated in the LC as well as treat or palliate *Diseases* localized in the LC (Supplementary Fig 5D). Similarly, Supplementary Figure 7E shows the connection between Anxiety (*SideEffect)* and the LC (*Anatomy)*. Interestingly, the shortest paths between Anxiety or Aggression to the LC only share three nodes: alcohol dependence, epilepsy syndrome, and hypertension. The final example shows the connections between fetal heart rate (*SideEffect)* and the umbilical artery (*Anatomy)* (Supplementary Fig. 5F). This connection is centered on a set of genes that are associated or regulated by Diseases localized in umbilical artery. Those same *Genes* are also targets of or regulated by *Compounds* that impact fetal heart rate. These examples further show that PSEVs can be used to find related biomedical entities and further our understanding of how and why they are connected.

# SUPPLEMENTARY FIGURES

### Supplementary Figure 1. Calculating non-uniform probability of random jump.

In the original PageRank paper, vector *E* that allows the random surfer (walker) to avoid sinks (such as cycles with no outgoing edges) by giving the walker the ability to jump randomly to any node in the network. Usually *E* is uniform ($\beta$ /N where $\beta$ = the probability of random jump (restart parameter or 1-damping factor) and N = number of nodes in the network). **(a)** Calculation of Vector *E*. Here the walker is only allowed to restart at the SEPs and the probability of starting at a given SEP is dependent on the patient cohort with that SEP. **(b)** Example of the walker on mock SPOKE. The walker is currently on the "eating behavior" node in SPOKE. Black edges connect to neighbor nodes (Gene A, Gene B, and Gene C) that are not SEPs. The colorful gradient edges connect the "eating behavior" node to the SEPs. **(c)** Calculation of final transition vector from the "eating behavior" node.

Supplementary Figure 1

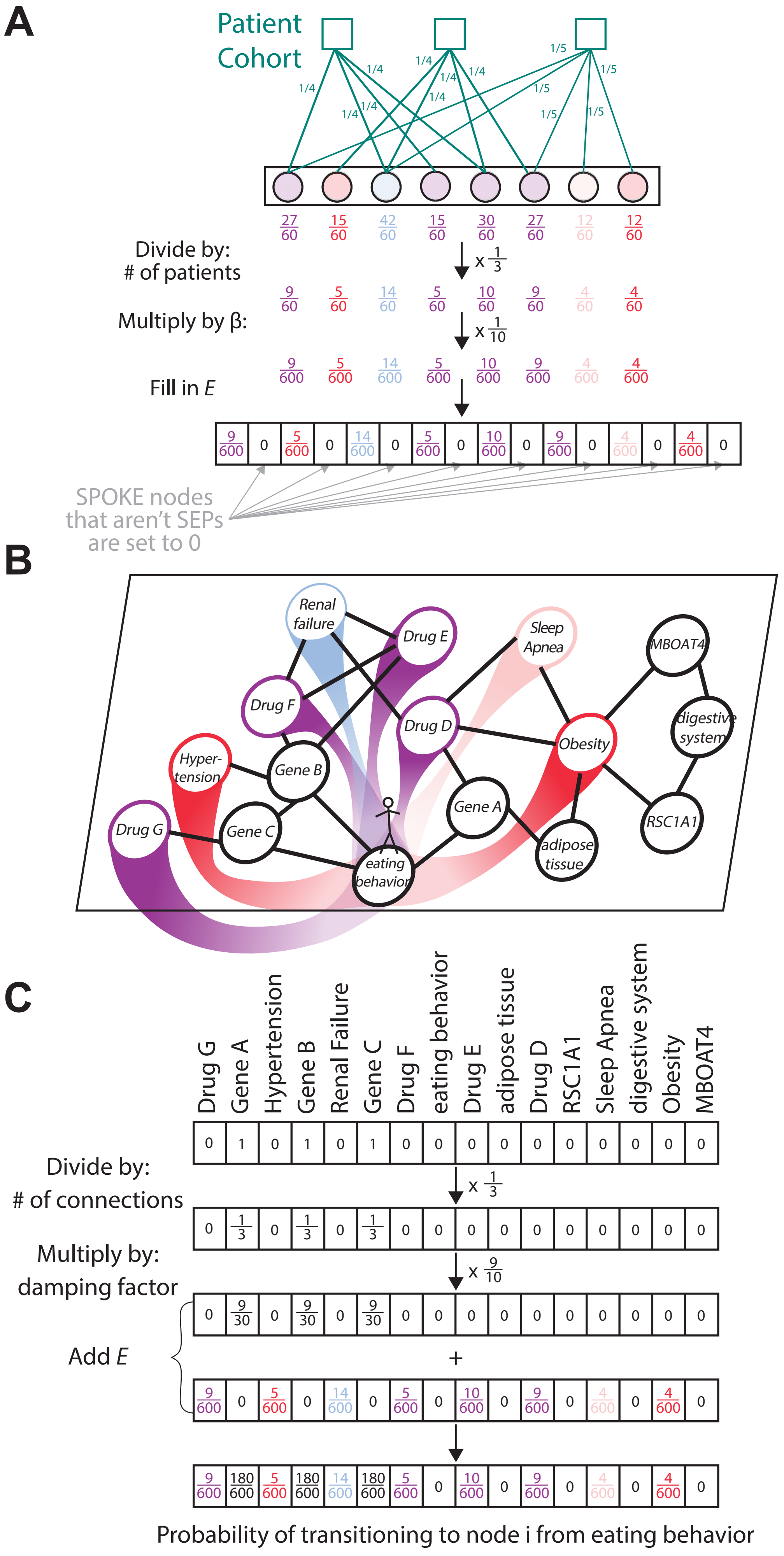

### Supplementary Figure 2. BMI PSEVs created using continuous BMI contain phenotypic and genotypic information.

PSEVs were created for cohorts of patients with BMI 15-50 (intervals of 1 BMI). **(a)** Continuous BMI vs *Disease* Rank. The top 4 ranked *Diseases* (obesity, hypertension, type 2 diabetes mellitus, and metabolic syndrome X) in Figure 2 still show a strong positive relationship with BMI when treating BMI as a continuous variable. The opposite trend also holds for celiac disease, Crohn's disease, and attention deficit disorder. **(b)** FTO gene was positively correlated with BMI.

Supplementary Figure 2

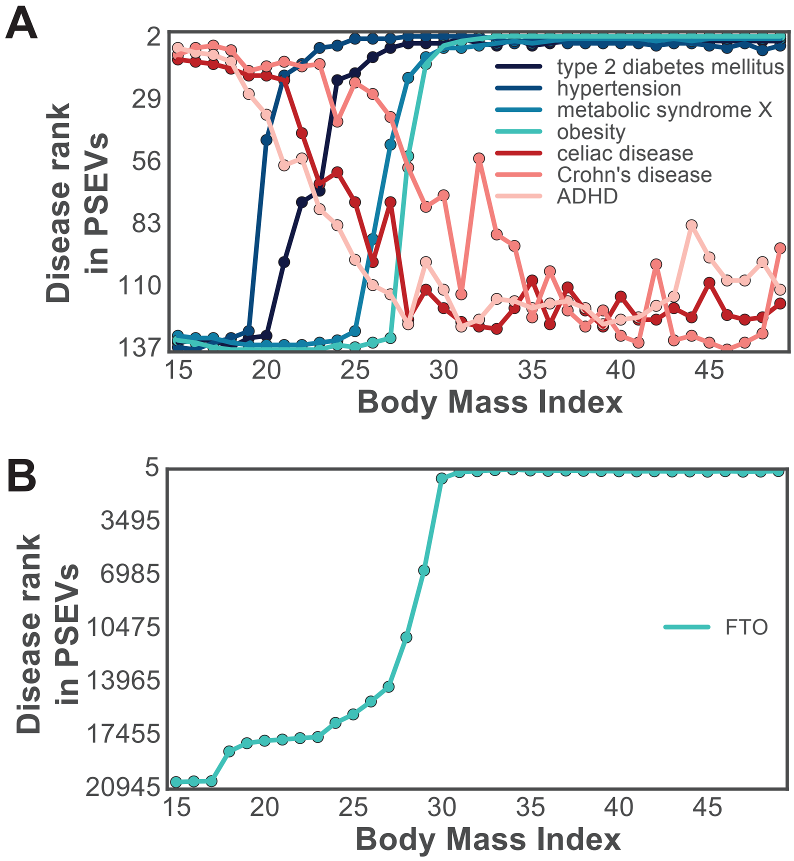

## Supplementary Figure 3. PSEVs embed first neighbors in SPOKE and learn new relationships.

(A) Distribution of ranks in PSEV vectors for first neighbors (blue) and non-first neighbors (red). (B) Multiple sclerosis first neighbors that overlap with top PSEV rank (blue edges) or not in top PSEV rank (red). (C) The top 10 ranked nodes in the PSEV for each node types that don't directly connect to Multiple sclerosis Disease node in SPOKE (dashed edges)

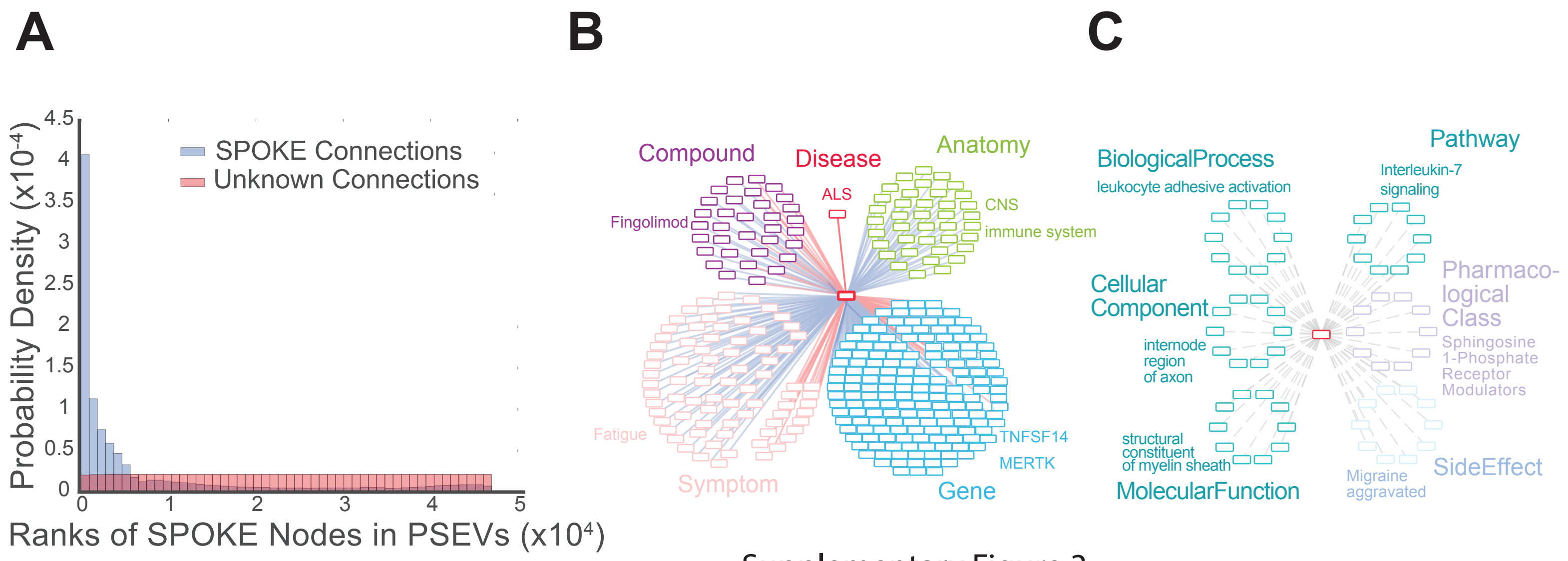


Supplementary Figure 3

## Supplementary Figure 4. Recovering deleted *Disease-Disease* edges.

**(A)** shows how the deleted *Disease-Disease* edge between Type 2 Diabetes and Hypertension was recovered using the pressure generated from the Type 2 Diabetes patients. **(B)** The gold standard *Disease-Disease* network was made from the deleted edges in SPOKE. Plots show the number of *Disease-Disease* relationships using each of the PSEV matrices that overlap with the gold standard network. The pink distributions show the results from the permuted PSEV matrices ($PSEV^{Random}$; 1000 iterations) while the arrows show the results from the original PSEV (blue), $PSEV^{\Delta DD, \Delta DG}$ (green), $PSEV^{SPOKE\ SHUFFLED}$ (red), and $PSEV^{SEP\ SHUFFLED}$ (orange). **(B)** The top K *Diseases* where selected from the set of *Diseases* in the gold standard network or **(B insert)** the entire set of *Disease* in SPOKE. **(F)** The top K *Diseases* where selected from the set of *Diseases* in the gold standard network or **(F insert)** the entire set of *Disease* in SPOKE. PD (Parkinson's disease).

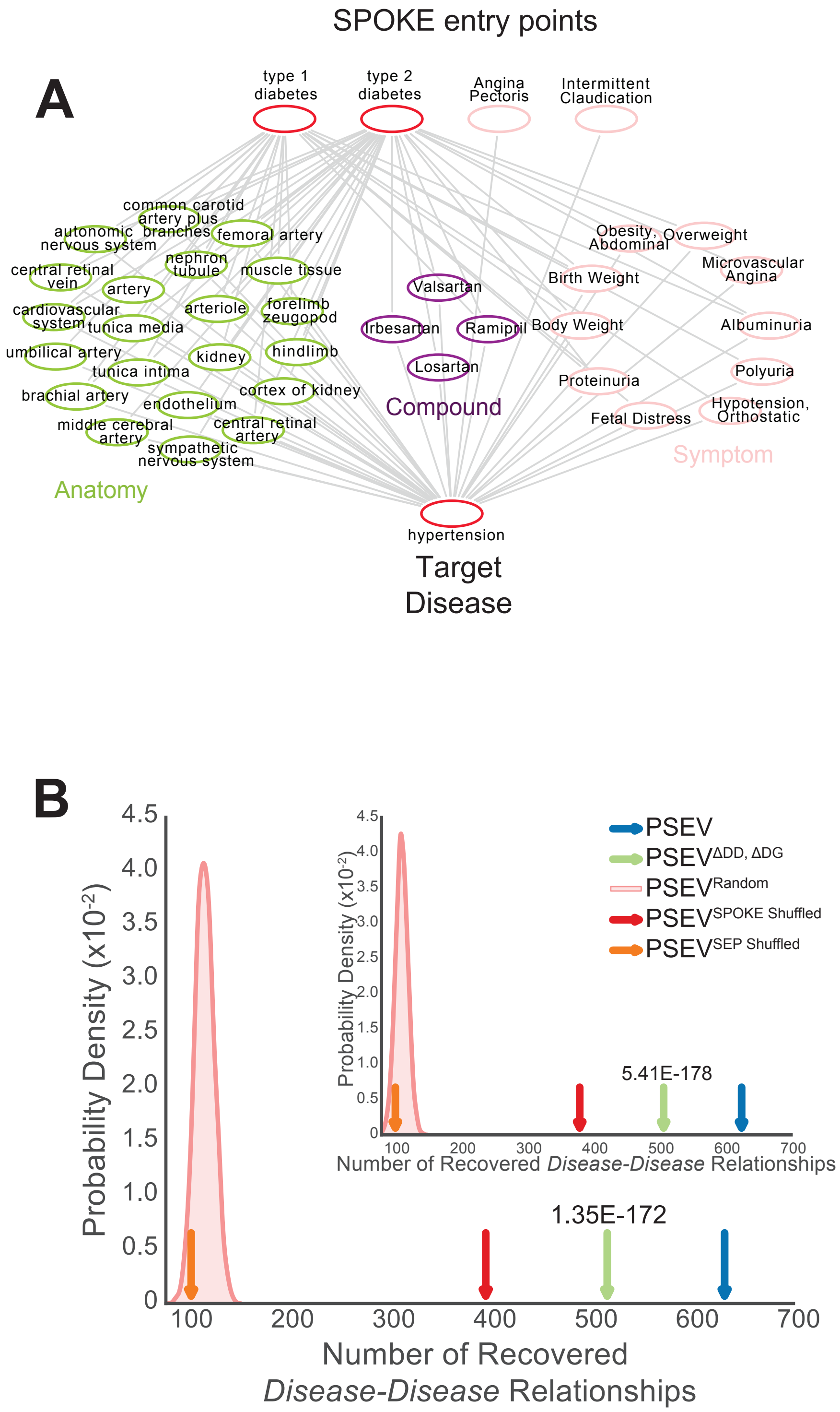


# Supplementary Figure 4

## Supplementary Figure 5. Recovering deleted *Compound-Gene* edges.

Prior to PSEV$^{\Delta CC, \Delta CG}$ calculation all of the *Compound -Gene* and *Compound - Compound* edges were deleted from SPOKE. It is possible to retrace how PSEV can recover deleted edges (outlined in Figure 4C). **(A)** Shortest paths between the top SEPs of Tolmetin, a non-steroidal anti-inflammatory drug, to its target *PTGS1*. **(B)** The gold standard *Compound-Gene* network was made from the deleted edges in SPOKE (*Compound*-BINDS_CbG-*Gene*). Plots show the number of *Compound-Gene* relationships using each of the PSEV that overlap with the gold standard networks. The pink distributions show the results from the permuted PSEV matrices (PSEV$^{Random}$; 1000 iterations) while the arrows show the results from the original PSEV (blue), PSEV$^{\Delta CC, \Delta CG}$ (green), PSEV$^{SPOKE\ SHUFFLED}$ (red), and PSEV$^{SEP\ SHUFFLED}$ (orange). **(B)** The top K *Genes* where selected from the set of *Genes* in the gold standard network or **(B insert)** the entire set of *Gene* nodes in SPOKE. **(C-E)** Determining K threshold for recovering *Compound-Gene* edges. **(C)** The top factor in determining missing *Compound-Gene* edges was whether patients that were on a given compound were also diagnosed with a Disease that was a associated with the target gene. **(D)** Shows the mean number of recovered *Compound-Gene* relationships at each rank (where 1=top ranked and 1451 was the worst ranked *Gene*; CI=95%). **(E)** Shows how much the patients that were prescribed a given *Compound* were contributing to the rank of the binding partner (missing *Compound-Gene* relationship) of that *Compound* using the flow of information through Diseases as in A (CI=95%). Genes ranked greater than ~150 were no longer receiving positive patient contribution. ADHD (Attention deficit hyperactivity disorder); AD (Alzheimer's disease), HT (hypertension), ES (epilepsy syndrome), SCZ (schizophrenia), D-TMP (Dexmethylphenidate),

# Supplementary Figure 5

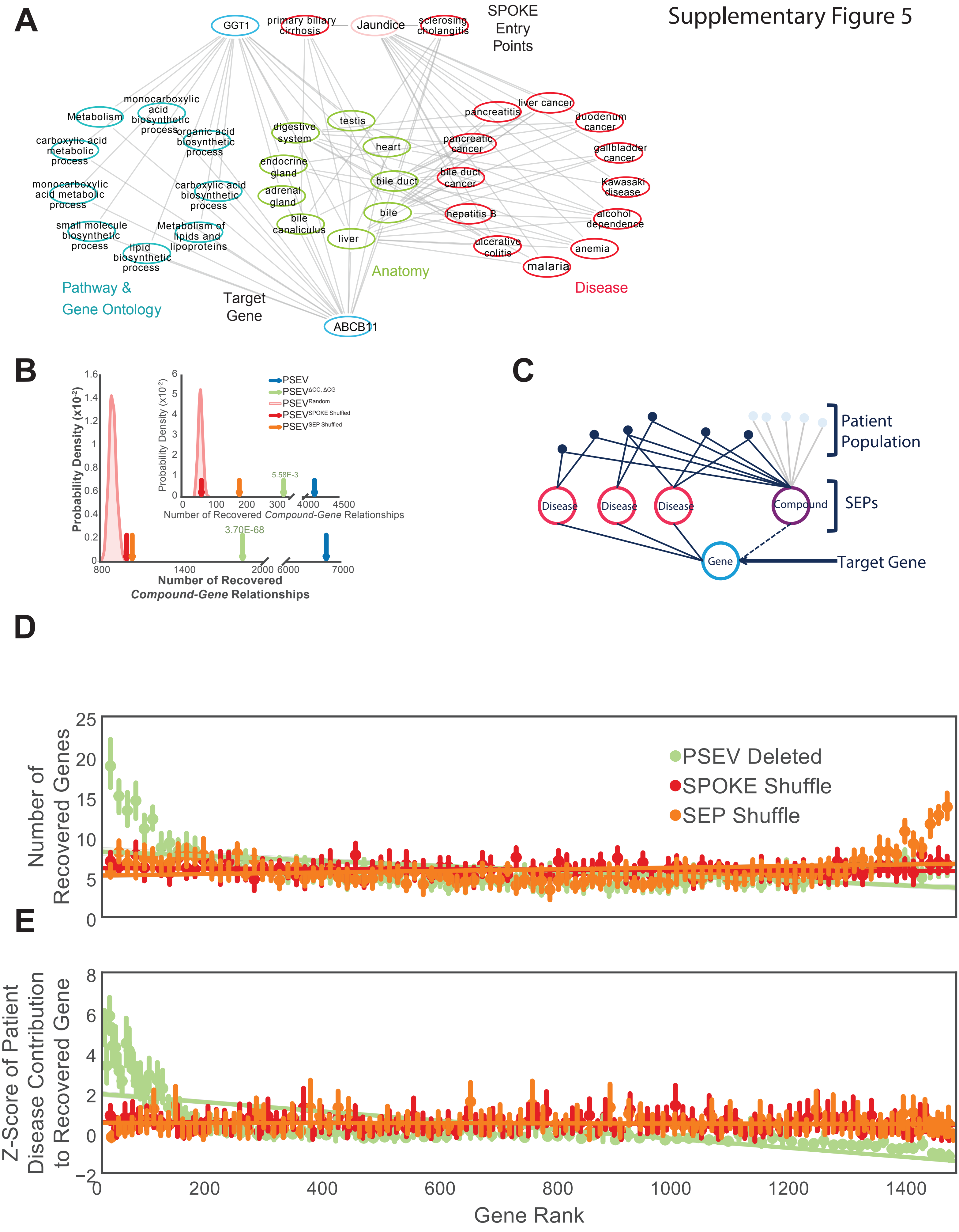

### Supplementary Figure 6. Recovering deleted *Compound-Compound* edges.

**(A)** Retracing shortest between similar *Compounds*. The paths between Tioconazole to Sertaconazole and Trihexyphenidyl to Procyclidine show two different routes in finding similar compounds. **(B)** The gold standard *Compound- Compound* network was made from the deleted edges in SPOKE (*Compound*-RESEMBLES_CrC-*Compound*). **(B)** The top K *Compound* where selected from the set of *Compound* in the gold standard network or **(B insert)** the entire set of *Compound* in SPOKE.

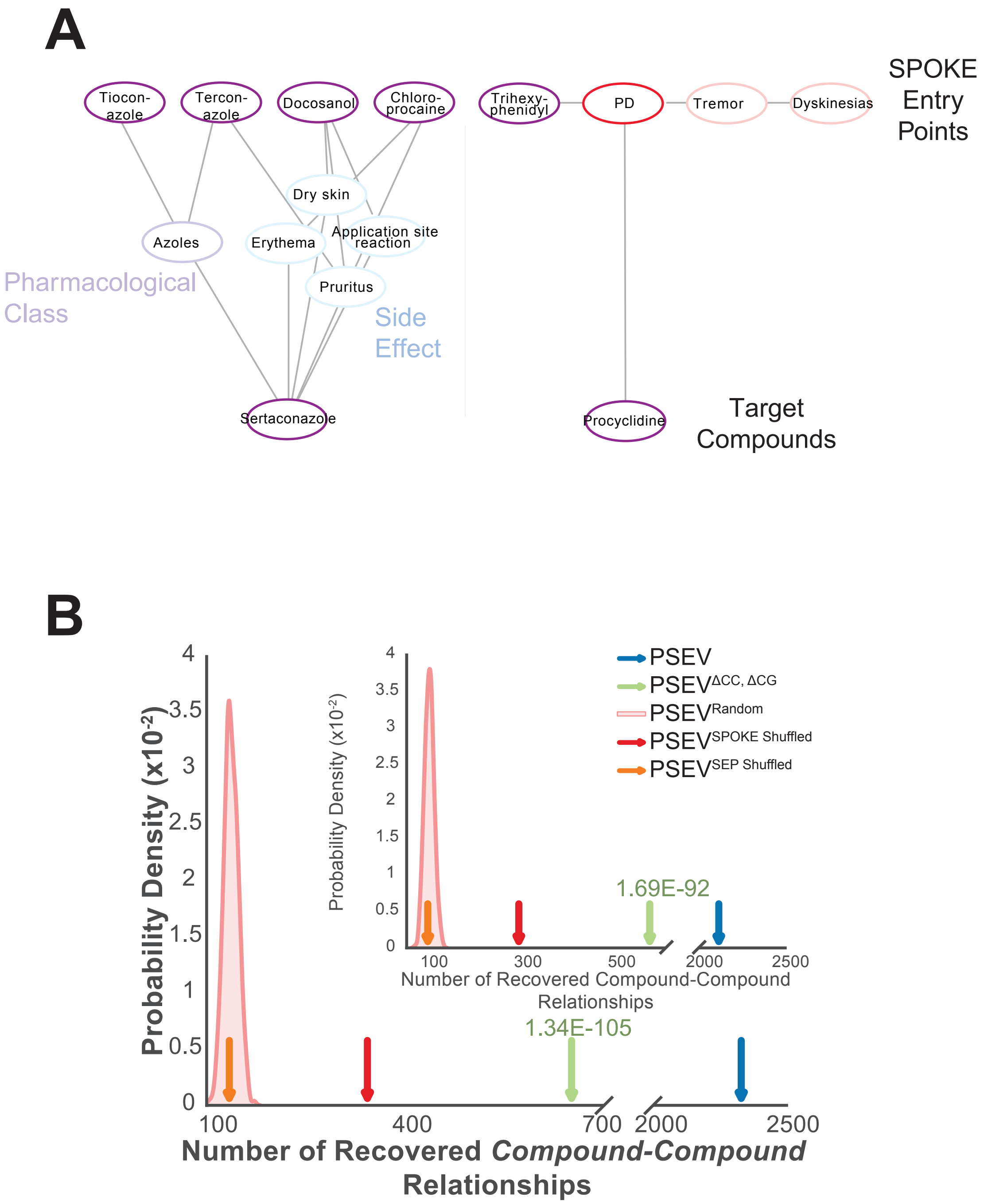


Supplementary Figure 6

## Supplementary Figure 7 MEDLINE Anatomy-SideEffect Relationships are Top Ranked Nodes in PSEV.

Fraction **(A),** count **(B)**, and fold change **(C)** of overlapping edges MEDLINE *Anatomy-SideEffect* network and PSEV *Anatomy-SideEffect* network (blue) or random PSEV *Anatomy-SideEffect* network (red) for different thresholds of PSEV disease similarity. A-C Are shown in 5% similarity intervals of ranked nodes starting with the most similar 5% left and all nodes (100%) right. The inserts in A-C focus on the top 0.14-1.6% of ranked nodes. Ribbon in (C) shows range of fold change for different values of $\beta$ (plots A-B use optimized $\beta$=0.1). D-F Examples shortest paths connecting the nodes that contribute the most to the *SideEffect-Anatomy* similarity to the target *SideEffect* and *Anatomy* nodes.

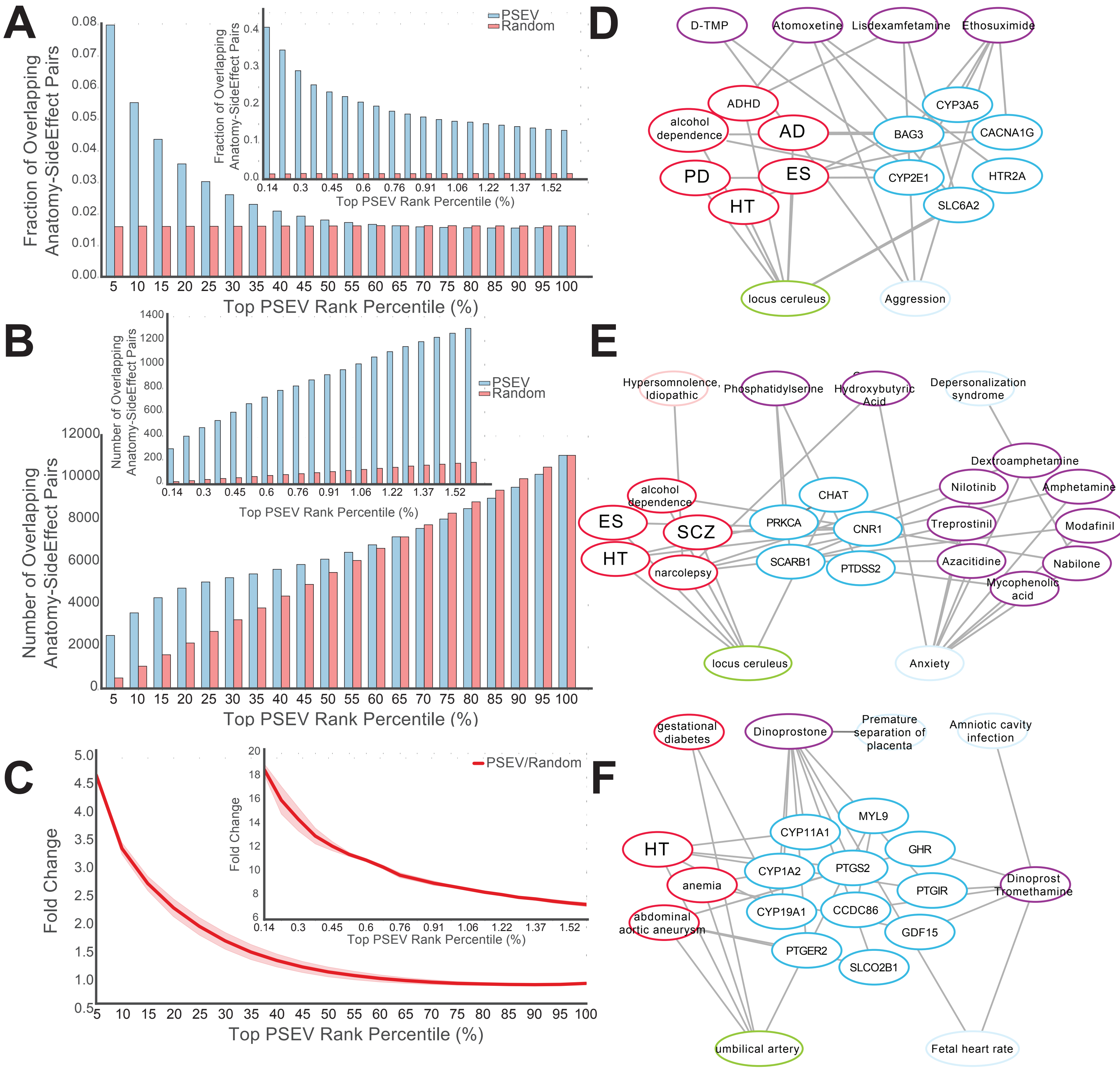


Supplementary Figure 7

## Supplementary Tables

Supplementary Table 1. **SPOKE nodes**. Source(s) and counts of each node type in SPOKE.

| Node Name | Source | Count |
|---|---|---|
| Gene | Entrez Gene | 20945 |
| BiologicalProcess | Gene Ontology | 11381 |
| SideEffect | UMLS via SIDER 4.1 | 5734 |
| MolecularFunction | Gene Ontology | 2884 |
| Compound | DrugBank | 1552 |
| CellularComponent | Gene Ontology | 1391 |
| Pathway | Reactome via Pathway Commons | 1308 |
| Symptom | MeSH | 438 |
| Anatomy | Uberon | 402 |
| PharmacologicClass | FDA via DrugCentral | 345 |
| Pathway | WikiPathways | 294 |
| Pathway | PID via Pathway Commons | 220 |
| Disease | Disease Ontology | 137 |
| Total | | 47031 |

Supplementary Table 2. **SPOKE edges.** Source(s) and counts of each edge label in SPOKE.

| Edge Name | Source | Count |
|---|---|---|
| DOWNREGULATES_AdG | Bgee | 102240 |
| UPREGULATES_AuG | Bgee | 97848 |
| RESEMBLES_CrC | Dice similarity of ECFPs | 6486 |
| INCLUDES_PCiC | DrugCentral | 1029 |
| COVARIES_GcG | ERC | 61690 |
| DOWNREGULATES_CdG | LINCS L1000 | 21102 |
| REGULATES_GrG | LINCS L1000 | 265672 |
| UPREGULATES_CuG | LINCS L1000 | 18756 |
| LOCALIZES_DlA | MEDLINE cooccurrence | 3602 |
| PRESENTS_DpS | MEDLINE cooccurrence | 3357 |
| RESEMBLES_DrD | MEDLINE cooccurrence | 543 |
| PARTICIPATES_GpBP | NCBI gene2go | 559504 |
| PARTICIPATES_GpCC | NCBI gene2go | 73566 |
| PARTICIPATES_GpMF | NCBI gene2go | 97222 |
| PALLIATES_CpD | PharmacotherapyDB | 390 |
| TREATS_CtD | PharmacotherapyDB | 755 |
| PARTICIPATES_GpPW | PID via Pathway Commons | 8154 |
| PARTICIPATES_GpPW | WikiPathways | 12587 |
| PARTICIPATES_GpPW | Reactome via Pathway Commons | 63631 |
| CAUSES_CcSE | SIDER 4.1 | 138944 |
| DOWNREGULATES_DdG | STARGEO | 7623 |
| UPREGULATES_DuG | STARGEO | 7731 |
| ASSOCIATES_DaG | DOAF, GWAS Catalog, DisGeNET, DISEASES | 12623 |
| BINDS_CbG | DrugBank (target), PDSP Ki, PubChem, DrugCentral (IUPHAR), ChEMBL, DrugCentral (label), BindingDB, DrugCentral (ChEMBL), DrugCentral (KEGG DRUG), DrugBank (enzyme), DrugCentral (literature), DrugCentral (ChEMBL, DrugBank (carrier), DrugBank (transporter), US Patent | 11571 |
| EXPRESSES_AeG | Bgee, TISSUES | 526407 |
| INTERACTS_GiG | Lit-BM-13, hetio-da, Venkatesan-09, Yu-11, hetio-dag, HI-II-14, HI-I-05, II_binary, II_literature | 147164 |
| **Total** | | **2250197** |

## Supplementary References


1. Himmelstein, D.S. *et al.* Systematic integration of biomedical knowledge prioritizes drugs for repurposing. *bioRxiv* **manuscript in evaluation in eLife**(2017).
2. Chen, X., Liu, M. & Gilson, M.K. BindingDB: a web-accessible molecular recognition database. *Comb Chem High Throughput Screen* **4**, 719-25 (2001).
3. Gilson, M.K. *et al.* BindingDB in 2015: A public database for medicinal chemistry, computational chemistry and systems pharmacology. *Nucleic Acids Res* **44**, D1045-53 (2016).
4. Law, V. *et al.* DrugBank 4.0: shedding new light on drug metabolism. *Nucleic Acids Res* **42**, D1091-7 (2014).
5. Wishart, D.S. *et al.* DrugBank: a comprehensive resource for in silico drug discovery and exploration. *Nucleic Acids Res* **34**, D668-72 (2006).
6. Ursu, O. *et al.* DrugCentral: online drug compendium. *Nucleic Acids Res* **45**, D932-D939 (2017).
7. Radi, Z.A. & Ostroski, R. Pulmonary and cardiorenal cyclooxygenase-1 (COX-1), -2 (COX-2), and microsomal prostaglandin E synthase-1 (mPGES-1) and -2 (mPGES-2) expression in a hypertension model. *Mediators Inflamm* **2007**, 85091 (2007).
8. Bruno, A., Tacconelli, S. & Patrignani, P. Variability in the response to non-steroidal anti-inflammatory drugs: mechanisms and perspectives. *Basic Clin Pharmacol Toxicol* **114**, 56-63 (2014).
9. Green, R.M., Hoda, F. & Ward, K.L. Molecular cloning and characterization of the murine bile salt export pump. *Gene* **241**, 117-23 (2000).
10. Schuetz, E.G. *et al.* Disrupted bile acid homeostasis reveals an unexpected interaction among nuclear hormone receptors, transporters, and cytochrome P450. *J Biol Chem* **276**, 39411-8 (2001).
11. Mita, S. *et al.* Vectorial transport of bile salts across MDCK cells expressing both rat Na+-taurocholate cotransporting polypeptide and rat bile salt export pump. *Am J Physiol Gastrointest Liver Physiol* **288**, G159-67 (2005).
12. Rogers, D. & Hahn, M. Extended-connectivity fingerprints. *J Chem Inf Model* **50**, 742-54 (2010).
13. Morgan, H. The generation of a unique machine description for chemical structures-a technique developed at chemical abstracts service. *Journal of Chemical Documentation* **5**, 107-113 (1965).
14. Dice, L.R. Measures of the Amount of Ecologic Association Between Species. *Ecology* **26**, 297-302 (1945).
15. Benarroch, E.E. The locus ceruleus norepinephrine system: functional organization and potential clinical significance. *Neurology* **73**, 1699-704 (2009).
