# Supplementary material for: Integrating biomedical research and electronic health records to create knowledge-based biologically meaningful machine-readable embeddings
Source: Nat Commun. 2019 Jul 10;10:3045. doi: 10.1038/s41467-019-11069-0 (PMC6620318; doi:10.1038/s41467-019-11069-0)
Supplement: Supplementary file 2 — Reporting Summary [file 41467_2019_11069_MOESM2_ESM.pdf]

nature research

Corresponding author(s): Sergio E Baranzini

Last updated by author(s): Jun 14, 2019

Reporting Summary

Nature Research wishes to improve the reproducibility of the work that we publish. This form provides structure for consistency and transparency in reporting. For further information on Nature Research policies, see Authors & Referees and the Editorial Policy Checklist.

Statistics

For all statistical analyses, confirm that the following items are present in the figure legend, table legend, main text, or Methods section.

n/a	Confirmed	
☐	☒	The exact sample size (*n*) for each experimental group/condition, given as a discrete number and unit of measurement
☒	☐	A statement on whether measurements were taken from distinct samples or whether the same sample was measured repeatedly
☐	☒	The statistical test(s) used AND whether they are one- or two-sided<br>*Only common tests should be described solely by name; describe more complex techniques in the Methods section.*
☒	☐	A description of all covariates tested
☐	☒	A description of any assumptions or corrections, such as tests of normality and adjustment for multiple comparisons
☐	☒	A full description of the statistical parameters including central tendency (e.g. means) or other basic estimates (e.g. regression coefficient) AND variation (e.g. standard deviation) or associated estimates of uncertainty (e.g. confidence intervals)
☒	☐	For null hypothesis testing, the test statistic (e.g. *F*, *t*, *r*) with confidence intervals, effect sizes, degrees of freedom and *P* value noted<br>*Give P values as exact values whenever suitable.*
☒	☐	For Bayesian analysis, information on the choice of priors and Markov chain Monte Carlo settings
☒	☐	For hierarchical and complex designs, identification of the appropriate level for tests and full reporting of outcomes
☒	☐	Estimates of effect sizes (e.g. Cohen's *d*, Pearson's *r*), indicating how they were calculated

Our web collection on statistics for biologists contains articles on many of the points above.

Software and code

Policy information about availability of computer code

Data collection	A dedicated SQL database was used to store all (de-identified) flat files from EMRs
Data analysis	Python and R was used to write all code.

For manuscripts utilizing custom algorithms or software that are central to the research but not yet described in published literature, software must be made available to editors/reviewers. We strongly encourage code deposition in a community repository (e.g. GitHub). See the Nature Research guidelines for submitting code & software for further information.

Data

Policy information about availability of data

All manuscripts must include a data availability statement. This statement should provide the following information, where applicable:

- Accession codes, unique identifiers, or web links for publicly available datasets
- A list of figures that have associated raw data
- A description of any restrictions on data availability

The version of SPOKE used in the research is available on neo4j [neo4j.het.io/browser] and github [https://github.com/hetio/hetionet]. The PSEVs are available on the PSEVexplorer [psevexplorer.ucsf.edu] and github [github.com/baranzini-lab/PSEV].

Field-specific reporting

Please select the one below that is the best fit for your research. If you are not sure, read the appropriate sections before making your selection.

☒ Life sciences ☐ Behavioural & social sciences ☐ Ecological, evolutionary & environmental sciences

For a reference copy of the document with all sections, see nature.com/documents/nr-reporting-summary-flat.pdf

Life sciences study design

All studies must disclose on these points even when the disclosure is negative.

Sample size	Almost a million records were used for this analysis. Power calculations were not performed, as we deemed sample size to be adequate.
Data exclusions	We selected only records from patients diagnosed with at least one of the 137 diseases currently present in our SPOKE database. Out of 878,479 patients 292,753 had at least one of the 137 complex diseases currently represented in SPOKE.
Replication	Independent replication was not attempted given the complexity and potential privacy concerns of this dataset (although completely de-identified). However, internal cross-validation was conducted by removing critical components of the network to assess robustness of our findings.
Randomization	Randomization of the network was performed in some of our analyses to establish robustness.
Blinding	Our experimental design did not allow for blinding. However, robustness was assessed by multiple-fold randomization

Reporting for specific materials, systems and methods

We require information from authors about some types of materials, experimental systems and methods used in many studies. Here, indicate whether each material, system or method listed is relevant to your study. If you are not sure if a list item applies to your research, read the appropriate section before selecting a response.

Materials & experimental systems

n/a	Involved in the study
☒	☐ Antibodies
☒	☐ Eukaryotic cell lines
☒	☐ Palaeontology
☒	☐ Animals and other organisms
☒	☐ Human research participants
☒	☐ Clinical data

Methods

n/a	Involved in the study
☒	☐ ChIP-seq
☒	☐ Flow cytometry
☒	☐ MRI-based neuroimaging
